# Extracellular vesicles from pluripotent stem cell-derived mesenchymal stem cells acquire a stromal modulatory proteomic pattern during differentiation

**DOI:** 10.1038/s12276-018-0142-x

**Published:** 2018-09-10

**Authors:** Alejandro La Greca, Claudia Solari, Veronica Furmento, Antonella Lombardi, Maria Celeste Biani, Cyntia Aban, Lucia Moro, Marcela García, Alejandra Sonia Guberman, Gustavo Emilio Sevlever, Santiago Gabriel Miriuka, Carlos Luzzani

**Affiliations:** 1 LIAN-CONICET, FLENI, Ruta 9 Km 52,5 – (B1625XAF), Belén de Escobar, Buenos Aires, Argentina; 2 Agencia Nacional de Promoción Científica y Tecnolígica (ANPCyT), Buenos Aires, Argentina; 3 Laboratorio de Regulación Génica en Células Madre, INQUIBICEN – CONICET, Departamento de Química Biológica, FCEyN, Universidad de Buenos Aires, Buenos Aires, Argentina; 4 Consejo Nacional de Investigaciones Científicas y Técnicas (CONICET), Buenos Aires, Argentina; 5 Cátedra de Citología, Histología y Embriología A, Facultad de Ciencias Médicas, Universidad Nacional de La Plata, Buenos Aires, Argentina

## Abstract

Mesenchymal stem/stromal cells (MSCs) obtained from pluripotent stem cells (PSCs) constitute an interesting alternative to classical MSCs in regenerative medicine. Among their many mechanisms of action, MSC extracellular vesicles (EVs) are a potential suitable substitute for MSCs in future cell-free-based therapeutic approaches. Unlike cells, EVs do not elicit acute immune rejection, and they can be produced in large quantities and stored until ready to use. Although the therapeutic potential of MSC EVs has already been proven, a thorough characterization of MSC EVs is lacking. In this work, we used a label-free liquid chromatography tandem mass spectrometry proteomic approach to identify the most abundant proteins in EVs that are secreted from MSCs derived from PSCs (PD-MSCs) and from their parental induced PSCs (iPSCs). Next, we compared both datasets and found that while iPSC EVs enclose proteins that modulate RNA and microRNA stability and protein sorting, PD-MSC EVs are rich in proteins that organize extracellular matrix, regulate locomotion, and influence cell–substrate adhesion. Moreover, compared to their respective cells, iPSCs and iPSC EVs share a greater proportion of proteins, while the PD-MSC proteome appears to be more specific. Correlation and principal component analysis consistently aggregate iPSCs and iPSC EVs but segregate PD-MSC and their EVs. Altogether, these findings suggest that during differentiation, compared with their parental iPSC EVs, PD-MSC EVs acquire a more specific set of proteins; arguably, this difference might confer their therapeutic properties.

## Stem cells: Proteins in secreted vesicles offer potential therapy

Tiny protein-containing vesicles released by partially differentiated stem cells contain a suite of therapeutic proteins that make them a promising alternative to cell-based treatments. Carlos Luzzani from LIAN-CONICET in Buenos Aires, Argentina, and colleagues reprogrammed skin cells to an embryonic-like state, and then coaxed these induced pluripotent stem cells (iPSCs) to form mesenchymal stem/stromal cells (MSCs), a type of adult stem cell that can give rise to bone, muscle and other tissues. The researchers analyzed all the proteins produced by the iPSCs, MSCs and the sub-micron sized bubbles known as extracellular vesicles that each secretes. They found that vesicles from MSCs, but not iPSCs, included a small set of proteins involved in regeneration and immune modulation. These vesicles may provide the regenerative benefits of MSCs without the safety risks of a cell-based therapy.

## Introduction

Mesenchymal stem/stromal cells (MSCs) are one of the most promising cell types in regenerative medicine. Because of their multilineage differentiation potential^1^ and immunological modulatory properties^2–5^, MSCs are currently being tested in more than 6900 clinical studies (www.clinicaltrials.gov, query: Mesenchymal stem cells OR Mesenchymal Stromal Cells OR MSCs, searched on December 2017). Initially, MSCs were believed to be recruited at the site of injury; however, it was later proposed that their therapeutic action was directly exerted via replacing the damaged tissue. Currently, in addition to direct differentiation, MSCs are believed to secrete a myriad of soluble factors and extracellular vesicles (EVs) that modulate the behavior of cells in a paracrine fashion^6–9^.

MSCs can be readily isolated from adult tissues such as the bone marrow, adipose tissue, and umbilical cord. However, MSC therapeutic properties decline rapidly in vitro with the number of passages^10,11^. This poses a substantial problem when expanding cells to obtain the amount required for clinical purposes. Recently, MSCs were obtained from pluripotent stem cells (PSCs)^12–14^. PSCs are mainly found in the inner cell mass from the blastocyst, called embryonic stem cells (ESCs); adult cells reprogrammed by forced expression of pluripotency transcription factors are called induced PSCs (iPSCs)^15^. PSCs are able to differentiate into endoderm, ectoderm, and mesoderm lineage, including MSCs. MSCs derived from PSCs (PD-MSCs) can differentiate into the osteogenic, chondrogenic, and adipogenic lineage; have surface markers such as CD90, CD73, and CD105; and have immunological modulatory properties that make them indistinguishable from patient-derived MSCs^16^.

The therapeutic properties of MSCs are explained, at least in part, by the paracrine action of EVs. EVs are classified mainly by their size and cargo^17,18^. These include apoptosomes, microvesicles, and the smaller exosomes. EVs are particularly important in explaining MSC regenerative features^8,19–21^, and since they mediate intercellular communication, they are considered key components of a potential cell-free, off-the-shelf therapy. MSC EVs are already being clinically tested for graft-versus-host disease and chronic kidney disease^22^. Additionally, PD-MSC EVs were found to protect against renal ischemia/reperfusion injury^23^, and they have multiple effects on cutaneous wound healing, bone regeneration, and hindlimb ischemia and vascular injury repair^24^.

EVs usually enclose lipids, mRNA, microRNAs (miRNAs), and proteins that upon recognition of their target cells are able to regulate their function. While microvesicles originate directly from shedding of the plasma membrane, exosomes are formed by invagination of a specific endosomal compartment called multivesicular bodies (MVBs). Exosomes are then released upon fusion of MVBs to the plasma membrane, and they either adhere to the membrane of target cells or are internalized by the latter, which induces specific signals^25^. Loading of cargo into exosomes is far from being a stochastic event. In particular, proteins can be directed to exosomes through endosomal sorting complexes required for transport (ESCRT)-dependent or ESCRT-independent mechanisms, where tetraspanins and the lipid composition of vesicles plays key roles^26–30^. In addition, ESCRT is also involved in the sorting of proteins to shedding vesicles such as microvesicles^31^.

Although there is an increasing amount of literature regarding the viability of PD-MSCs as a robust source of MSCs, little is known regarding the content of their secretome. In particular, in this work, we sought to gain insight regarding the proteomic content of their EVs and aimed to further characterize them with the objective of improving the chances of their future application in cell-free-based therapy.

## Materials and methods

### Cell culture

WA09 human embryonic stem cells were purchased from WiCell (Madison, WI, USA). iPSCs were generated in our laboratory as previously described^32^. Briefly, foreskin fibroblasts were reprogrammed by transfection with the STMCCA lentivirus, which was generously obtained from Gustavo Mostoslavsky^33^. Several clones have been characterized in our laboratory via demonstration of their pluripotent state, its ability to differentiate into cells from the three germinal layers, and the formation of teratomas. For this paper, we have used the clone FN2.1. PSCs were cultured in E8-defined medium over vitronectin-coated plates. PD-MSCs were differentiated in our laboratory as previously published^16^. Briefly, on day 0 of differentiation, PSCs were incubated with Accutase until the cells were completely dissociated, and they were plated onto Geltrex-coated plates and suspended in α-minimum essential medium (α-MEM) supplemented with platelet lysate 10% and B27 1/100 (all from Life Technologies, Carlsbad, CA, USA). A ROCK inhibitor (Y27632 10 ng/ml; Tocris, Avon, Bristol, UK) was added every time the cells were passaged until day 14 of differentiation. From that day onwards, the cells were passed on plastic dishes with no coating and were grown in medium supplemented with PL 10% and penicillin–streptomycin. Wharton jelly (WJ)-MSCs were grown in α-MEM supplemented with PL 10%. All experiments were performed using MSCs maintained in culture until passage ten or less.

### EV isolation and transmission electron microscopy

EVs were isolated according to Théry et al. ^34^. Briefly, cells were seeded to reach 80% of confluence. The cells were then cultured for 24 h in E8-defined medium (Life Technologies). The cell culture supernatants were then centrifuged at 4 °C, 2000 x *g* for 10 min, which was followed by another centrifugation at 4 °C, 10,000 x *g* for 30 min and one last ultracentrifugation at 4 °C, 100,000 x *g* for 90 min. The EVs were the washed and ultracentrifuged again. The pellet was then resuspended in phosphate-buffered saline (PBS) or RIPA Buffer (Sigma-Aldrich, St. Louis, MO, USA) without protease inhibitors and was stored at −80 °C, depending on the experiment to be performed. The resuspended EVs were quantified via the Pierce BCA Protein Assay (Thermo Scientific, Waltham, MA, USA). Transmission electron microscopy (TEM) was performed according to Théry et al.^34^ by the LANAIS-MIE Core Facility at the IBCN Institute, University of Buenos Aires/CONICET (National Research Council) using a Zeiss EM 109T with a Gatan ES1000W digital camera.

### Flow cytometry analysis

Flow cytometry analyses were performed in a BD Accuri cytometer. First, 20 µg of EV resuspension was bound to 20 µl of anti-CD63 antibody-coated magnetic beads (Life Technologies), as recommended by the manufacturer. Next, a second staining with anti-CD81-PE-conjugated antibody or anti-CD9-APC-conjugated antibody (Molecular Probes, Life Technologies, Eugene, OR, USA) was performed for 30 min at room temperature. The beads were then washed with PBD plus albumin 0.1% and were analyzed. At least 5000 events per treatment were counted.

### Mass spectrometry analysis

Liquid chromatography tandem mass spectrometry (LC-MS/MS) was performed on three biological replicates of each type of EVs and one biological replicate of each type of cell. The datasets were then grouped according to the EV or cell type. Each group was run at least three times. Protein digestion and mass spectrometry analysis were performed at the Proteomics Core Facility CEQUIBIEM, at the University of Buenos Aires/CONICET (National Research Council) as follows: the protein samples were reduced with dithiothreitol in 50 mM of ammonium bicarbonate at a final concentration of 10 mM (45 min, 56 °C) and were alkylated with iodoacetamide in the same solvent at a final concentration of 20 mM (40 min, room temperature (RT), in darkness). This protein solution was precipitated with 1/5 volumes of trichloroacetic acid at −20 °C for at least 2 h and was centrifuged at maximum speed for 10 min (4 °C). The pellet was washed twice with cool acetone and was dried at RT. The proteins were resuspended in ammonium bicarbonate 50 mM, pH = 8, and were digested using trypsin (V5111; Promega, Madison, WI, USA). Next, the peptides were purified and desalted via ZipTip C18 columns (Millipore, Burlington, MA, USA).

The digests were analyzed via nanoLC-MS/MS in a Thermo Scientific Q-Exactive Mass Spectrometer coupled to a nanoHPLC EASY-nLC 1000 (Thermo Scientific). For the LC-MS/MS analysis, approximately 1 μg of peptides was loaded onto the column and was eluted for 120 min using a reversed-phase column (C18, 2 µm, 100 A, 50 µm x 150 mm) Easy-Spray Column PepMap RSLC (P/N ES801) that was suitable for separating protein complexes with a high degree of resolution. The flow rate used for the nanocolumn was 300 nl min^−1^, and the solvent range used was from 7% B (5 min) to 35% B (120 min). Solvent A was 0.1% formic acid in water, whereas B was 0.1% formic acid in acetonitrile. The injection volume was 2 µL. The MS equipment has a high collision dissociation cell (HCD) for fragmentation and an Orbitrap analyzer (Q-Exactive, Thermo Scientific). A voltage of 3.5 kV was used for electrospray ionization (Thermo Scientific, EASY-SPRAY).

XCalibur 3.0.63 software (Thermo Scientific) was used for data acquisition and equipment configuration that allows peptide identification simultaneously with their chromatographic separation. Full-scan mass spectra were acquired in the Orbitrap analyzer. The scanned mass range was 400–1800 *m*/*z*, at a resolution of 70,000 at 400 *m*/*z*, and the 12 most intense ions in each cycle were sequentially isolated, fragmented by HCD, and measured in the Orbitrap analyzer. Peptides with a charge of +1 or with unassigned charge state were excluded from fragmentation for MS2.

### Analysis of MS data

Q-Exactive raw data was processed using Proteome Discoverer software (version 2.1.1.21, Thermo Scientific) and was searched against *Homo sapiens* protein sequence database with trypsin specificity and a maximum of one missed cleavage per peptide. Carbamidomethylation of cysteine residues was set as a fixed modification, and oxidation of methionine was set as variable modification.

Proteome Discoverer searches were performed with a precursor mass tolerance of 10 ppm and product ion tolerance of 0.05 Da. Static modifications were set to carbamidomethylation of Cys, and dynamic modifications were set to oxidation of Met and N-terminal acetylation. Protein hits were filtered for high confidence peptide matches with a maximum protein and peptide false discovery rate of 1%, which was calculated by employing a reverse database strategy.

Proteome Discoverer calculates an area for each protein in each condition. For this process, it uses the area under the curve of the three most intense peptides for a protein. The areas were calculated for each of the three triplicates and were normalized. The data obtained for the area for each protein were processed via the Perseus program (Max Planck Institute of Biochemistry, 1.5.5.3 version, available for free) that allows a deeper statistical analysis. Different scatter plots were prepared according to the compared samples. For each couple of samples, we plotted Log *p* value (−Log Student's *T* test *p* value A_B) on the *y*-axis versus Student's *T* test Difference A_B on the *x*-axis. Proteins that appear in the volcano plot with a fold change >2 (<−1 or >1 on the *x*-axis of the graph) and a *p* value below 0.05 (above 1.3 on the *y*-axis of the graph) were considered differentially expressed.

### Wound healing assay

Human microvascular endothelial cells (HMEC-1) were kindly provided by Dr. Gabriela Fernandez from IMEX-CONICET, Academia Nacional de Medicina. The cells were seeded to confluency in 12-well culture plates with MCDB (Molecular, Cellular and Developmental Biology; Thermo Scientific) medium, 10% fetal bovine serum, 10 ng/ml epidermal growth factor, and 1 µg/ml hydrocortisone, and after 18 to 20 h, injuries (wounds) were performed manually using a micropipette tip. Immediately after this, the cells were washed with PBS, and the injuries were photographed to register the initial setting (time zero: 0 h), followed by the addition of α-MEM medium containing or not containing (without EVs) EVs collected from PD-MSCs, WJ-MSCs, and iPSCs. After 22 h, the injuries were photographed to mark the conclusion of the experiment. Images were acquired via EVOS (Life Technologies) and were analyzed using ImageJ’s MRI Wound Healing Tool (ImageJ macros, open source software – Redmine repository).

### Bioinformatic analysis

Quantitation of protein abundance was performed using the areas calculated by Proteome Discoverer normalized by the sum of the areas of all the proteins detected in each run. We further validated this quantitation by comparing it to their emPAI value^35^ and performing a Spearman's correlation analysis (Supplementary Figure 1). Bioinformatic analysis was performed in R. Gene Ontology Enrichment (Biological Process) using Bioconductor DOSE^36^ and cluster.Profiler^37^ packages and an online web-based gene analysis toolkit (Cellular Compartments and node graph)^38^. Violin plots, heat maps, and principal component analysis (PCA) were plotted using ggplot2 package. Differential expression analysis was conducted with the Bioconductor DESeq^39^ package, using a spectral count-based quantification (peptide to spectral matching (PSM))^40^ as input, and evaluation of the enriched signature genes was performed with the gene set enrichment analysis (GSEA) algorithm^41^, employing 1000 permutations and default parameters.

## Results

### Characterization of EVs isolated from cell lines

**Fig. 1:**
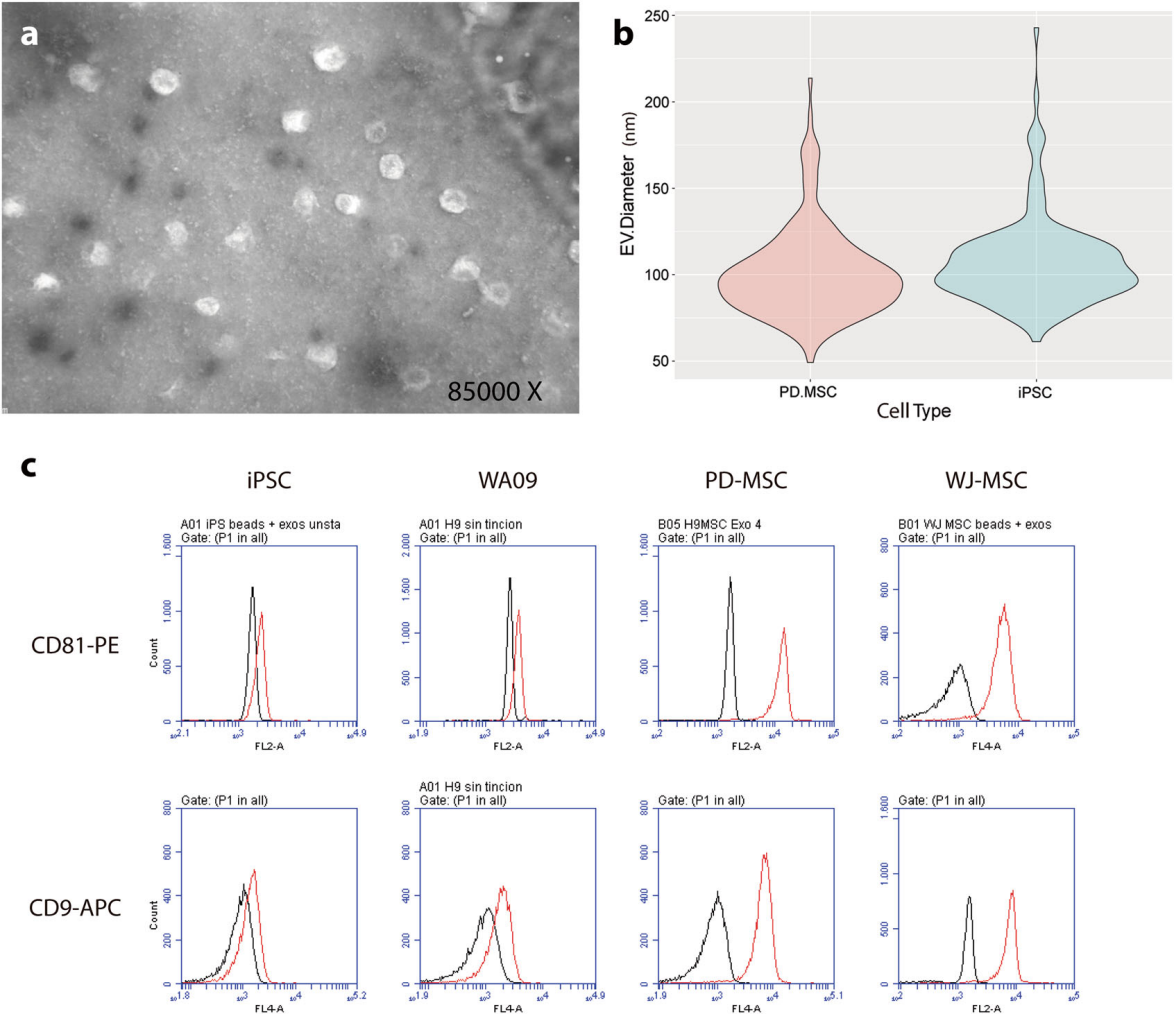
Characterization of EVs isolated from cell lines. **a** Transmission electron microscopy (TEM) of PD-MSC. **b** Quantification of diameters of PD-MSC EVs (left violin plot) and iPSC EVs (right violin plot) using ImageJ. **c** Flow cytometry analysis of EVs from different cell lines. After isolation, EVs were bound to anti-CD63-coated magnetic beads and were stained either with a CD81-PE-conjugated or a CD9-APC-conjugated antibody

Although their biogenesis, cargo, and function may widely differ, EVs remain mainly classified according to their size. TEM of iPSCs and PD-MSC EVs prepared in this work revealed vesicles of approximately 107 ± 12 and 101 ± 15 nm, respectively. As Fig. 1a, b show, these dimensions are compatible with small microvesicle and exosome sizes. However, the size distribution of PD-MSC EVs appears to be slightly more homogeneous than that of iPSC EVs, indicating that the latter might be composed of more than one type of EVs (Fig. 1b). In addition, EVs isolated from PSCs (iPSCs or WA09) and MSCs (derived from pluripotent cells (PDs) or from the umbilical cord’s WJ) exhibit the exosomal markers CD63, CD81, and CD9 (Fig. 1c; Supplementary Table 1). Intriguingly, pluripotent cells display a considerably lower intensity of CD9 and CD81 markers; however, the saturation of anti-CD63 magnetic beads was similar in all cases (data not shown).

### PD-MSC EV protein content exhibits stromal-related functions

**Fig. 2:**
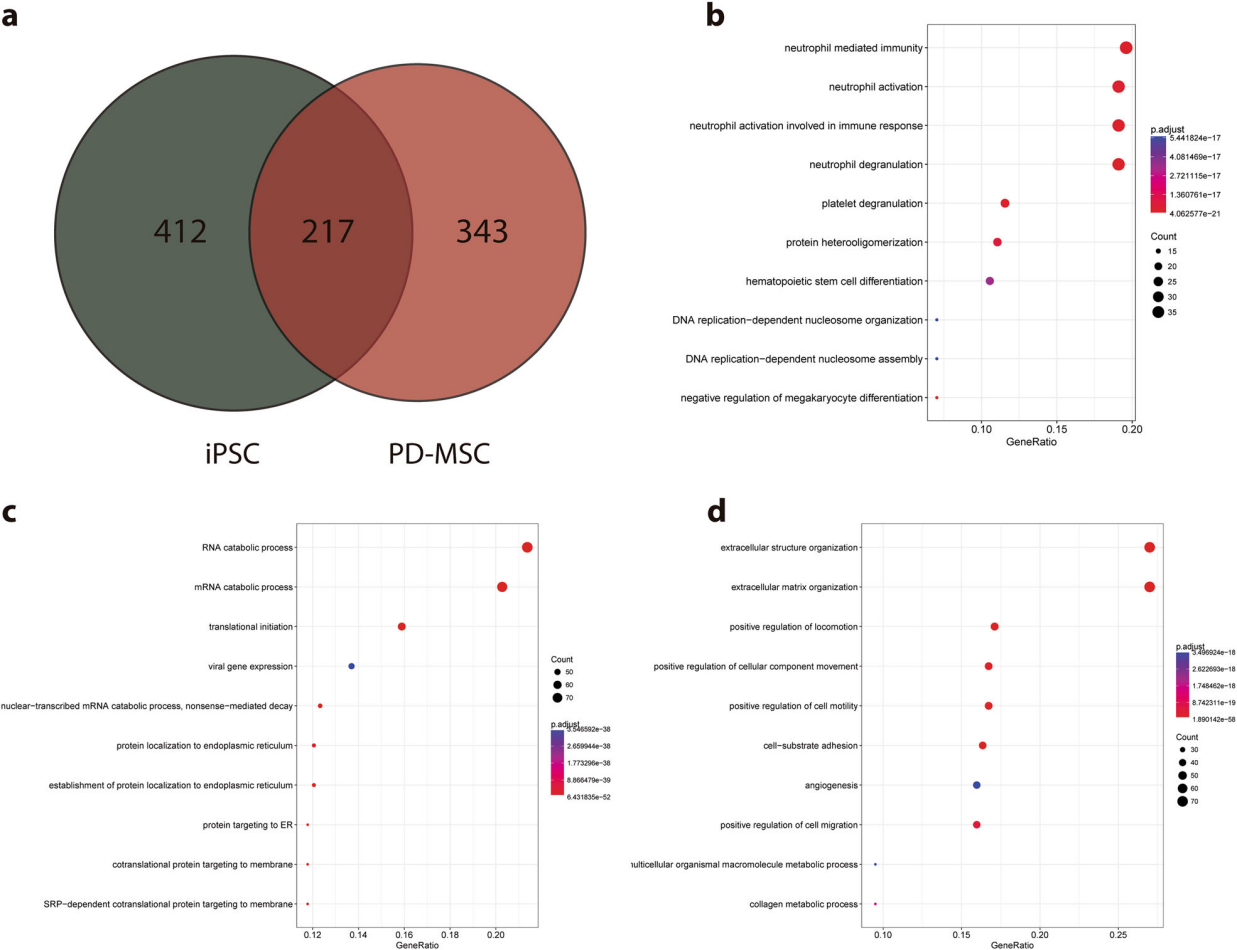
Comparison between iPSC EVs and PD-MSC EVs. **a** Venn diagram of UniProt IDs identified by LC-MS/MS. **b** Enriched Gene Ontology analysis (Biological Process) of shared UniProt IDs. **c** Enriched Gene Ontology analysis (Biological Process) of iPSC EV exclusive UniProt IDs. **d** Enriched Gene Ontology analysis (Biological Process) of PD-MSC EV exclusive UniProt IDs

As the number of approved MSC-based therapies rises, so does concern regarding the source of isolation of these cells and the scale of culture needed to meet future demands. Hence, direct differentiation of iPSCs into MSCs constitutes an interesting alternative source of MSCs. In this work, we aimed to characterize PD-MSC-EV proteome and compare it to the proteome of EVs from the iPSC line from which PD-MSCs were derived in the first place. Using LC-MS/MS analysis, we determined 629 unique UniProt IDs in iPSC EVs and 560 in PD-MSC EVs. When compared with each other, only 217 of these proteins were found to be shared (Fig. 2a). Gene Ontology Enrichment Analysis showed distinct biological processes for proteins that are either specific for iPSCs (Fig. 2c and Supplementary Table 2) or PD-MSCs (Fig. 2d and Supplementary Table 3) or are shared by both (Fig. 2b and Supplementary Table 4). Interestingly, while both types of EVs share proteins related to immune functions, EVs secreted by PD-MSCs differ greatly from those secreted by their isogenic iPSCs. As expected, vesicles from PD-MSCs mostly reflect stromal functions and extracellular matrix maintenance, suggesting a role of these EVs over the mesenchymal niche. Moreover, the unique protein cargo of iPSC EVs proved to be enriched in more general cellular processes such as RNA and miRNA catabolism, translational initiation, or protein targeting to the endoplasmic reticulum.

### PD-MSC EVs differ from their cell of origin more than iPSC EVs

**Fig. 3:**
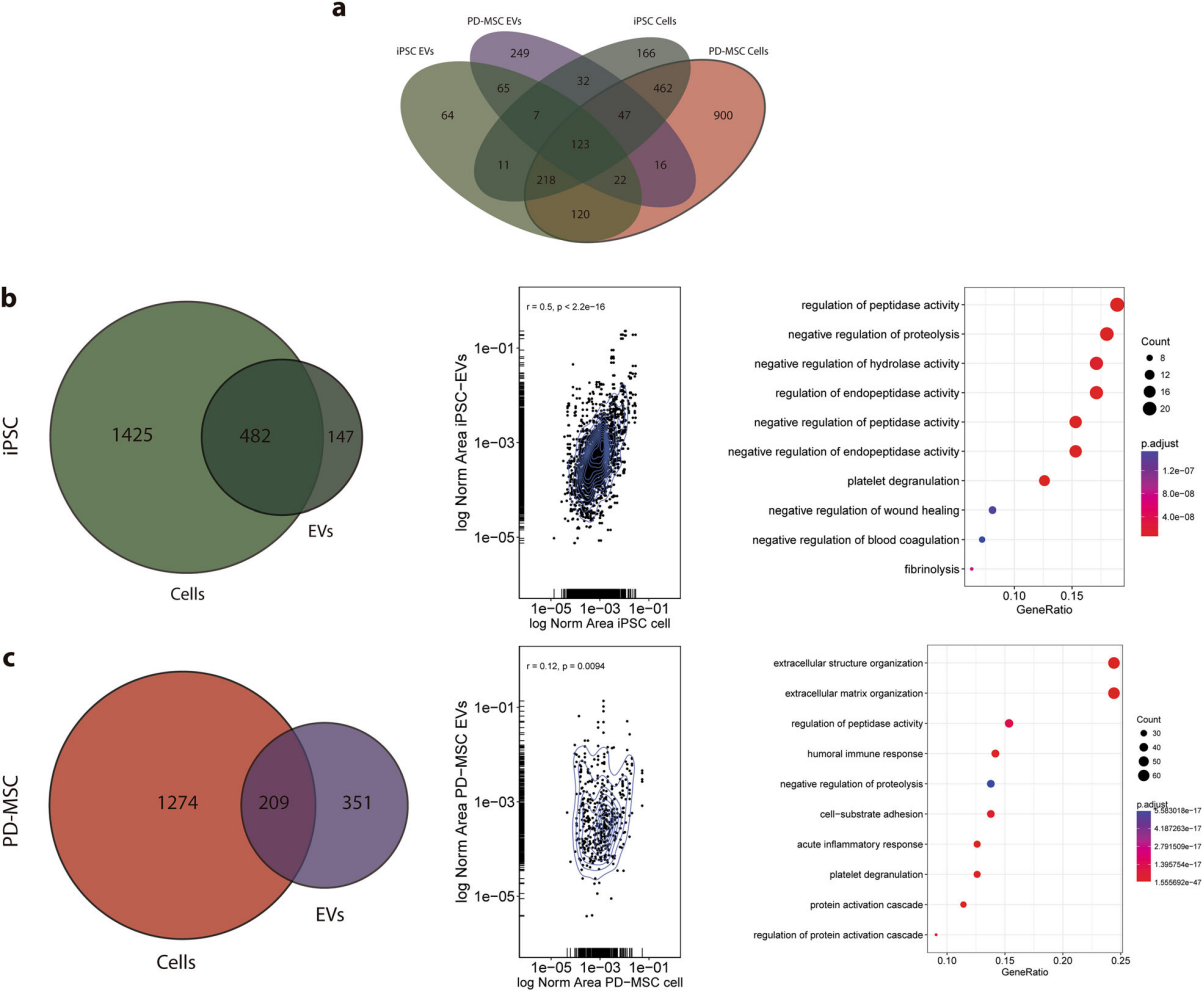
Comparison between EVs and the cells that secreted them. **a** QuadVenn diagram of UniProt IDs identified by LC-MS/MS. **b**, Left panel: Venn diagram of UniProt IDs of iPSCs versus their EVs. Middle panel: scatter plot of relative abundance of shared proteins between iPSCs and their EVs. Right panel: Enriched Gene Ontology analysis (Biological Process) of iPSC EV exclusive proteins. **c**, Left panel: Venn diagram of UniProt IDs of PD-MSCs versus their EVs. Middle panel: scatter plot of relative abundance of shared proteins between PD-MSCs and their EVs. Right panel: Enriched Gene Ontology analysis (Biological Process) of PD-MSC EV exclusive proteins

A major question regarding exosomal biogenesis is whether their cargo is selected actively, or in contrast, if it represents a mere reflection of the cell’s cytoplasm. A number of laboratories have contributed to unveil the mechanism of exosomal biogenesis; however, several of these experiments were conducted in different cell lines, leading to diverse and sometimes contradictory models. Taking advantage of the differentiation process to obtain MSCs from iPSCs, we sought to undertake this matter by comparing the proteome of each type of EV to the proteome of the cells that secreted them. Our rationale is that because PD-MSCs are differentiated directly from iPSCs, they constitute isogenic yet very different types of cells; therefore, their EVs might show different behaviors when compared to their respective cells. Figure 3 shows the most abundant proteins from iPSCs and their EVs (1907 and 629 unique UniProt IDs, respectively) (Fig. 3b), as well as from PD-MSCs and their EVs (1483 and 560 unique UniProt IDs, respectively) (Fig. 3c). The analysis showed that while iPSC vesicles share 76.63% of proteins with iPSCs, EVs secreted by PD-MSCs share only 37.32% of the proteins. In addition, scatter plots of the abundance of shared proteins show a higher correlation between iPSC EVs and their cells (Spearman: *r* = 0.5, *p* < 2.2e^−16^) rather than between PD-MSC EVs and their cells (Spearman: *r* = 0.12, *p* < 0.0094), suggesting a closer identity between the former. Enriched Gene Ontology analysis of proteins present in the EVs but not in the cell line that secreted them showed that iPSC EVs are rich in peptides that regulate proteolytic activity (Fig. 3b, right panel and Supplementary Table 5), while PD-MSC EVs appeared to be enriched in immune, extracellular matrix, and cell adhesion-controlling molecules (Fig. 3c, right panel and Supplementary Table 6).

### EVs segregate together in PCA and Pearson correlation assay

**Fig. 4:**
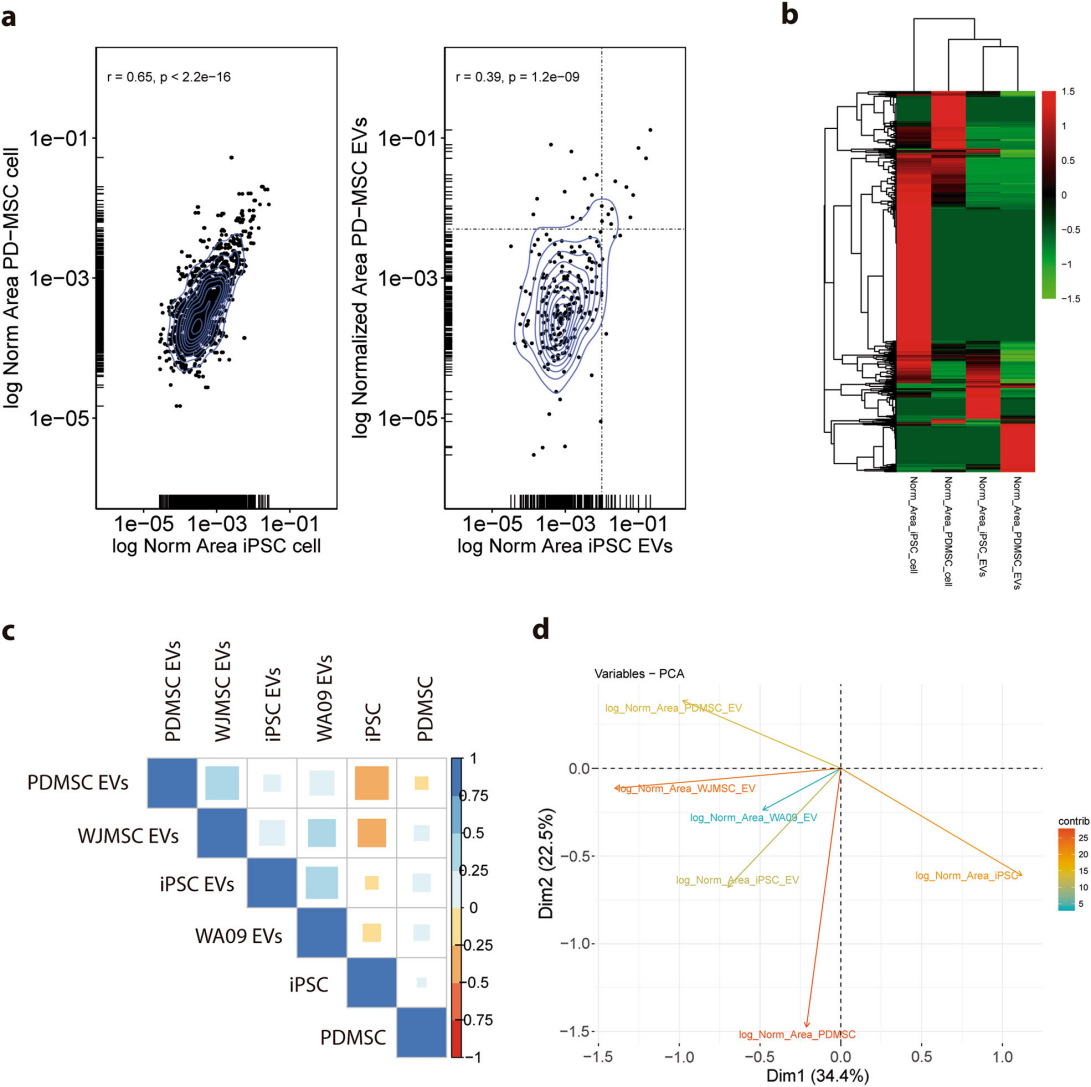
Clustering and abundance analysis of LC-MS/MS data. **a** Scatter plot showing abundance of shared proteins of iPSC versus PD-MSCs (left panel) and iPSC EVs versus PD-MSC EVs (right panel). **b** Heat map of protein abundance of iPSC and PD-MSC cellular and vesicular proteins. **c** Pearson's correlation assay of iPSCs and PD-MSCs and iPSC, PD-MSC, WA09, and WJ-MSC EV proteome. **d** Principal component analysis (PCA) of the same groups as in **c**

To explore the idea that PD-MSC EVs differ more with respect to their original cell than iPSC EVs, we first compared the abundance of shared proteins between the iPSC and PD-MSC lines and compared this result to the abundance of shared proteins between their EVs (Fig. 4a). Notably, the proteins in common between the cell types appeared to be detected at similar levels for both, suggesting a similar abundance within the cell (Fig. 4a, left panel). The same was not observed for EVs. Shared proteins between EVs of both cell types showed a greater dispersion in abundance, including proteins that were enriched preferably in iPSC EVs or in PD-MSC EVs; this indicated that during the differentiation process, packing of certain proteins may be altered (Fig. 4a, right panel). Proteins that were overrepresented in iPSC EVs, PD-MSC EVs, or both are detailed in Supplementary Table 7. The heatmap analysis, as presented in Fig. 4b, confirmed a defined cluster of overrepresented proteins for each group; however, some overlap is appreciated between groups. Interestingly, unsupervised clustering was performed on the heatmap group together with cellular datasets on one side and EV datasets on the other. Finally, we included in our analysis LC-MS/MS data from EVs secreted by umbilical cord-MSCs (WJ-MSCs) and the pluripotent line WA09, which are two related but not isogenic groups. We performed PCA (Fig. 4c) and plotted the two principal variables (56.9% of variance explained; Supplementary Figure 2), which showed certain dispersion of variables. However, the EV datasets were again grouped together, including WA09 EVs and WJ-MSC EVs. Moreover, the heat map, which showed abundance of proteins for all EVs, evidenced some degree of overlap (Supplementary Figure 3). Additionally, iPSC and PD-MSC cellular datasets showed different components, but were grouped together and apart from the EV datasets (Fig. 4d). This finding is again reinforced by the Pearson's correlation analysis, as displayed in Fig. 4d, where a positive correlation can be observed between all EV datasets; however, this correlation is greater among EVs of the same type (i.e., obtained from pluripotent (iPSCs and WA09) or multipotent (PD-MSC or WJ-MSC) stem cells; Supplementary Figure 4).

### EV protein content from MSCs reflects regenerative and immunomodulatory potential

**Fig. 5:**
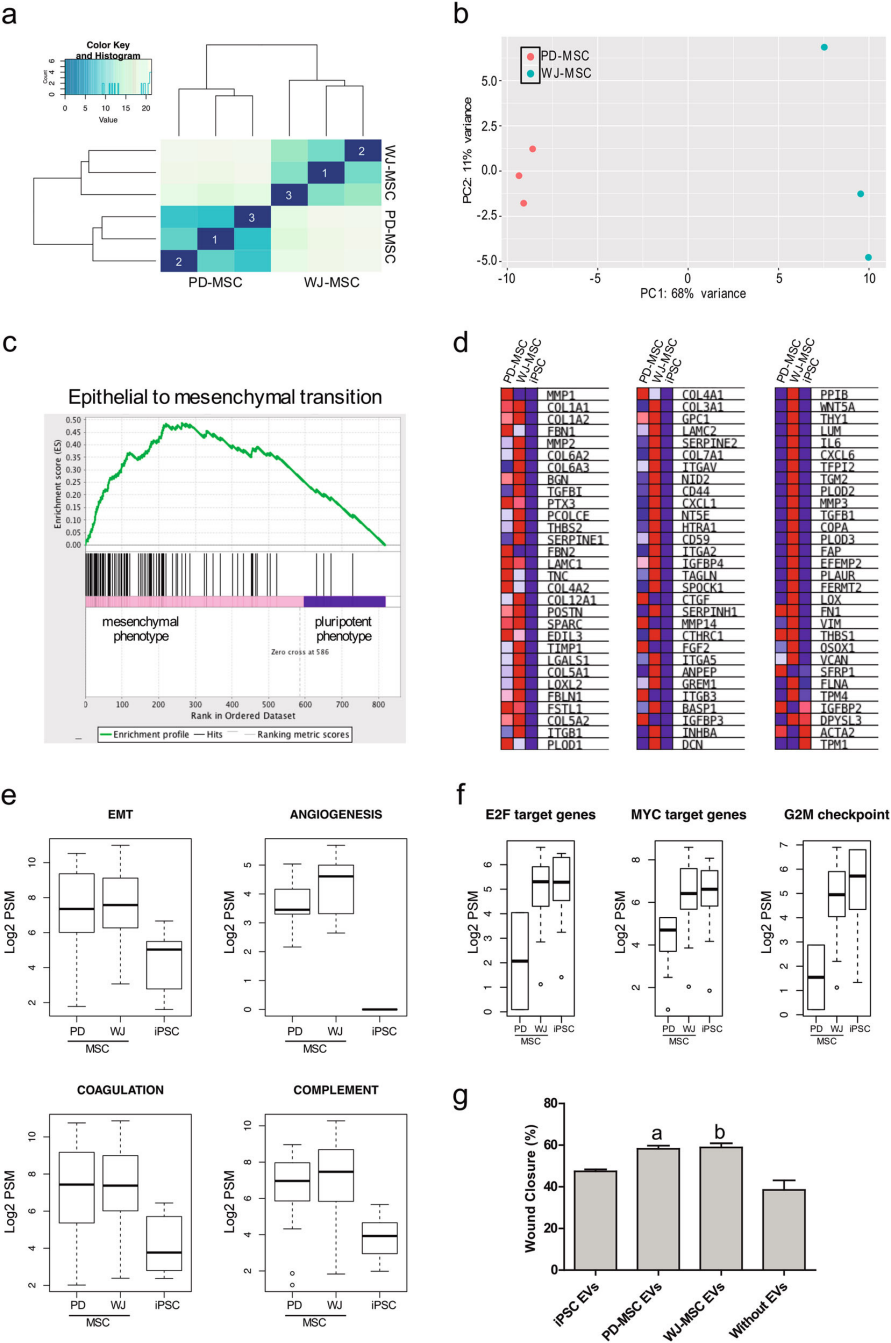
Functional analyses of EV content from PD-MSCs and WJ-MSCs. **a** Correlation analysis of proteins in EVs from PD-MSCs and WJ-MSCs. Matrix was plotted using three replicates for each EV source. **b** PCA for samples shown in **a**. **c** GSEA profile of EV protein content from PD-MSCs, WJ-MSCs, and iPSCs against epithelial-to-mesenchymal transition (EMT) signature genes. Proteins originated from both MSC EVs (PD and WJ) were assigned a mesenchymal phenotype, while proteins from iPSC EVs were assigned a pluripotent phenotype. **d** Heat map showing relative expression levels of proteins enriched in EMT GSEA plot (**c**). Red indicates high abundance, and blue indicates low-to-no abundance. **e**, **f** Distribution of normalized spectral counts expressed as Log 2 in four different sets of signature genes related to regeneration (**e**) and three related to cell cycle regulation (**f**) for EVs originated from PD-MSCs, WJ-MSCs, and iPSCs. **g** Wound healing assay results expressed as the mean percentage (%) of wound closure relative to time zero in two independent experiments ± SE. **a** versus Without EVs (*p* value <0.05); **b** versus Without EVs (*p* value <0.01)

Initial assessment of the MS/MS data showed that the proteome identified in EVs that were originated from PD-MSCs and WJ-MSCs is similar (Fig. 4 and Supplementary Figure 3). Further analysis using a differential expression of a spectral count-based (PSM^37^) approach revealed minor differences between PD-MSC and WJ-MSC EVs, which evidenced a higher inter-replicate and lower inter-sample correlation (Fig. 5a). PCA corroborated this observation (68% of variance explained; Fig. 5b). Although extensively validated by many authors, the use of spectral counts for the estimation of differences in peptide abundance was verified in our datasets by studying whether PSM scores were linearly related to their corresponding area value (Supplementary Figure 5A). In addition, the distribution of PSM scores of all peptides contained in EVs from PD-MSCs, WJ-MSCs, and iPSCs were compared to avoid biased conclusions as a result of substantial predefined differences in counts between samples (Supplementary Figure 5B). Differential analysis of proteins contained in EVs from PD-MSCs and WJ-MSCs showed few proteins that were significantly (*p* < 0.01) more abundant in one type of EV with respect to the other (Supplementary Figure 5C); this result confirmed our preliminary evidence regarding the identity and quantity of proteins present in these two types of EVs.

Functional analysis of proteins contained in EVs from both types of MSCs demonstrates that the majority of them are part of the cell’s vesicle-mediated secretome (Supplementary Figure 6A), and their functions relate to important biological processes such as angiogenesis, modulation of immune responses, and platelet degranulation (Supplementary Figure 6B). To reinforce our findings, we employed the GSEA^41^ algorithm to contrast the two types of MSC-originated EVs to iPSC-originated EVs (mesenchymal versus pluripotent phenotype) and confirmed that the epithelial-to-mesenchymal transition (EMT) signature genes (Molecular Signatures database v6.1, GSEA^41^) are preferentially associated with EVs from MSCs (Fig. 5c). Coherently, the proteins enriched in this category correspond to collagen, matrix metalloproteases, and integrins, among others (Fig. 5d). Furthermore, signature gene sets as part of angiogenic, coagulatory, and complement processes were significantly concordant with the mesenchymal phenotype as well (Fig. 5e). In contrast, proteins contained in EVs from iPSCs mainly associated with cell cycle regulation signature gene sets such as targets of E2F and MYC transcription factors and G2M checkpoint regulators (Fig. 5f), advocating a mere reflection of the ability of pluripotent cells to renew themselves. Interestingly, EVs from WJ-MSCs evinced potential cell cycle regulation functions as well. Finally, we performed in vitro corroboration of our informatic analyses by exposing HMEC-1 cells to environments enriched with different EV types obtained from PD-MSCs, WJ-MSCs, and iPSCs (Fig. 5g). Significant wound closure relative to time zero (a versus Without EVs: *p* < 0.05; b versus Without EVs: *p* < 0.01) was detected only for MSC-originated EVs (Fig. 5g and Supplementary Figure 6C), suggesting that these microvesicles could potentially regenerate injured endothelial tissue.

## Discussion

Mesenchymal stem cells are being increasingly used in regenerative and immunomodulatory therapies. As the number of clinical trials and marketed products that utilize MSC increases, issues regarding the scaling and validation of cell production will also increase. In particular, age and condition of donors and a limited expansion span in culture^10,11^ challenge the academic community to find new and creative ways to produce MSCs or mimic their therapeutic properties. In recent years, different groups have described numerous protocols to obtain MSCs from PSCs^12–14^. In the past, our laboratory has focused on conducting this process in an inexpensive and clinical manner^16^. A major promise of MSCs is the use of their exosomes as therapeutic vectors since cellular therapies have several drawbacks that a small vesicle such as the exosome does not have. Particularly, PD-MSC exosomes have already proven to be effective in ameliorating some pathologies^42^. However, literature on characterization of PD-MSC-derived EVs is scarce. Here, we took advantage of the comparison of PD-MSC EVs with the EVs secreted by the cells from which they were derived—the iPSC.

In this work, we showed that EVs secreted from PD-MSCs and WJ-MSCs are rich in extracellular matrix, cell–substrate adhesion, and locomotion and cell motility proteins. Moreover, EVs from PD-MSCs and iPSCs share cell cycle regulation and DNA replication proteins; however, intriguingly, these proteins appeared to be better represented in WJ-MSCs than in PD-MSCs. In contrast, iPSC EVs appeared to be richer in proteins that regulate basic cellular functions including RNA and miRNA catabolism and protein trafficking. These findings led us to believe that while iPSC EVs more resemble the cell cytoplasm, PD-MSC EVs may have a more stromal-directed function. EVs play a key role in the modulation of the immune response against tumors^43,44^, induction of angiogenesis^45^, and cell invasion and metastasis^46^. In this sense, the profile of the protein composition of PD-MSC EVs and their ability to increase the rate of injury repair explains why they are potential suitable candidates for tissue regeneration.

To test the hypothesis that iPSCs change the relative composition of proteins of their EVs when they differentiate to MSCs, we compared EV proteomes to the ones belonging to their respective cells. We found that, effectively, iPSC EVs shared a greater proportion of proteins with iPSCs than PD-MSC EVs do with PD-MSCs. In addition, when analyzing the relative abundance of these shared proteins, there appeared to be a better correlation for iPSCs than for PD-MSCs. In addition, when correlation or PCA are applied to datasets, EVs were grouped together, while the cells were consistently plotted apart. However, the cellular identity of the EVs was maintained, because iPSCs and WA09 EVs tended to segregate together on the one hand, while PD-MSCs and WJ-MSCs tended to be grouped together on the other. Altogether, these evidence suggest that iPSC EVs have a cargo that more resembles the cell cytoplasm than does the cargo from PD-MSC EVs. Upon differentiation of iPSCs towards MSCs, PD-MSC EVs may acquire a more specific set of proteins that, allegedly, have a more specific effect over the stromal cell niche.

This study focused on the proteomic content of EVs. However, the vesicular cargo includes mRNA and miRNA that contribute greatly to their bystander effect. It is possible that the proteins enclosed in EVs have a more immediate effect on target cells, while the RNA has a somewhat retarded influence on cellular behavior. In addition, the finding that iPSC EVs have a richer content of proteins that modulate RNA stability contributes to the idea that there might be some sort of interaction between proteomic and transcriptomic EV cargo.

It is now known that protein cargo is not randomly loaded into exosomes. Exosomal proteins are enclosed in vesicles through ESCRT-dependent and ESCRT-independent mechanisms^27^. Moreover, the biogenesis of exosomes is also controlled by tetraspanins and lipids^27,29^. Additionally, the biogenesis and composition of exosomes seem to be modulated according to the cell type and the environmental cues, suggesting that different subsets exist for these EVs produced within the same cell. Although we cannot ensure that the proteomic analysis in this work involves only one type of EV, the homogeneity of sizes and the presence of CD63, CD9, and CD81 markers suggest that exosomes are an integral part of isolated EVs. Despite this, it is possible that differential protein composition in iPSC EVs and PD-MSC EVs is explained by the existence of an entirely distinct set of EVs that are secreted by one or the other cell type rather than a change in composition of a particular type of EV. Nevertheless, we believe that having an isogenic model that presents changes in its secretome throughout the differentiation process might be a powerful tool to understand exosome biology, thus bringing us one step closer to using exosomes as a therapeutic vector.

## Electronic supplementary material

Supplementary Figure Legends

Supplementary Figure 1

Supplementary Figure 2

Supplementary Figure 3

Supplementary Figure 4

Supplementary Figure 5

Supplementary Figure 6

Supplementary Table 1

Supplementary Table 2

Supplementary Table 3

Supplementary Table 4

Supplementary Table 5

Supplementary Table 6

Supplementary Table 7
