# Supplementary material for: Extracellular vesicles from pluripotent stem cell-derived mesenchymal stem cells acquire a stromal modulatory proteomic pattern during differentiation
Source: Exp Mol Med. 2018 Sep 10;50(9):119. doi: 10.1038/s12276-018-0142-x (PMC6131549; doi:10.1038/s12276-018-0142-x)
Supplement: Supplementary file 1 — Supplementary Figure Legends [file 12276_2018_142_MOESM1_ESM.doc]

Supplementary Figure Legends

Supplementary Figure 1: Scatter plot and Spearman correlation analysis of emPAI vs normalized area quantitation. Left panel: iPSC data; right panel: PD-MSC data.

Supplementary Figure 2: Scree plot of PCA analysis shown in figure 4. Variable variances are explained in six dimensions. The two first dimensions represent approximately 60% of total variance.

Supplementary Figure 3: Heat map of protein abundance of iPSC, WA09, PD-MSC and WJ-MSC EVs.

Supplementary Figure 4: Scatter plot and Spearman correlation analysis of protein abundance in EVs from WJ-MSCs vs PD-MSCs.

Supplementary Figure 5: Spectral counts and differential expression validation. a) Linear regression modeling of spectral counts (PSM) vs normalized area in three replicates of EV protein content from both WJ- (left panel) and PD-MSCs (right panel) cells. b) PSM distribution of all proteins identified in EVs from PD-MSCs, WJ-MSCs and iPSCs expressed as log2. c) MA plot originated from differential expression (DE) analysis using a p-value cut-off of 0.01. Red dots indicate statistically significant differences.

Supplementary Figure 6: Gene ontology analysis and wound healing assay. a,b) Protein IDs found in EVs from both PD- and WJ-MSCs were categorized by cellular compartment (a) and GO term (b). Enriched GO terms are shown in red boxes. c) Images of scratches performed in cultured HMEC cells at 0 and 22 Hs of exposure to EVs originated from iPSCs, PD-MSCs and WJ-MSCs. Results were compared to HMEC cells that were not exposed to EVs (Without EVs).
