# Supplementary figures and images for: Extracellular vesicles from pluripotent stem cell-derived mesenchymal stem cells acquire a stromal modulatory proteomic pattern during differentiation
Source: Exp Mol Med. 2018 Sep 10;50(9):119. doi: 10.1038/s12276-018-0142-x (PMC6131549; doi:10.1038/s12276-018-0142-x)

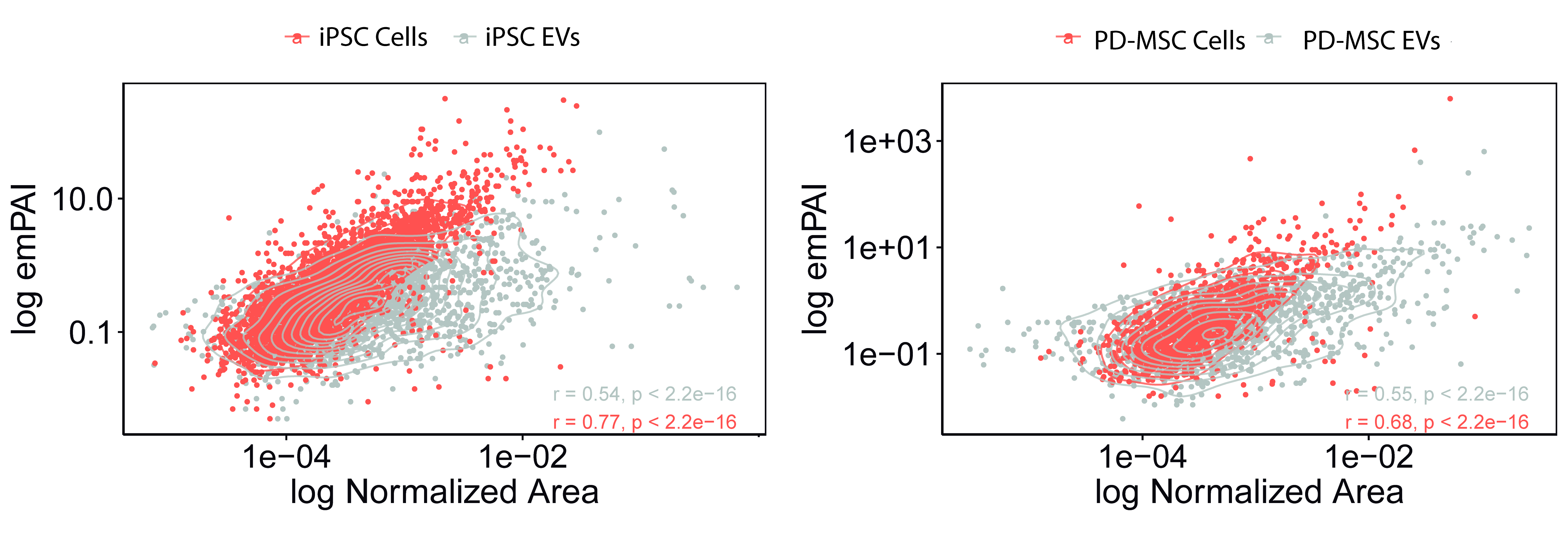

Supplement: Supplementary file 2 — Supplementary Figure 1 [file 12276_2018_142_MOESM2_ESM.tif]

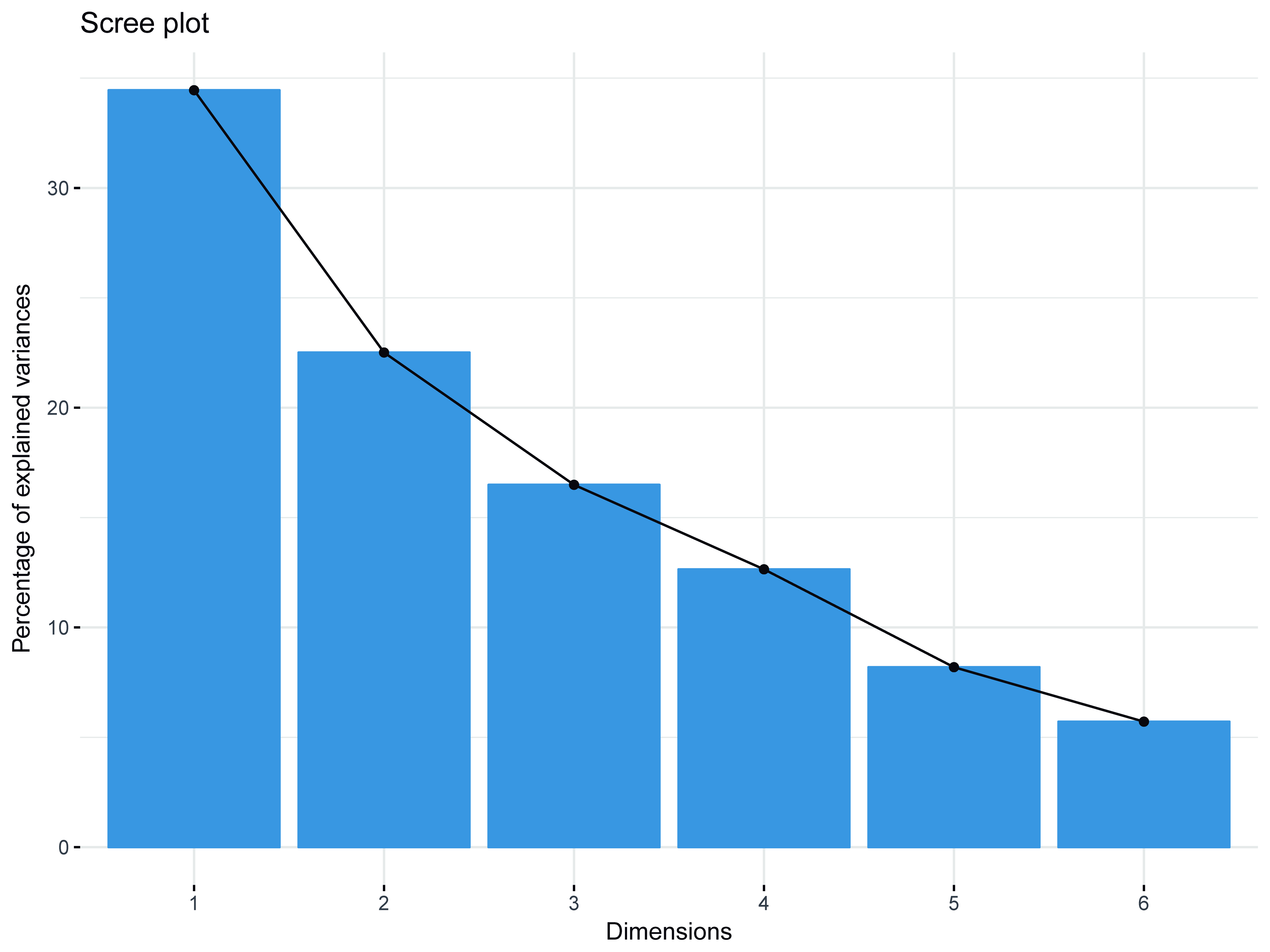

Supplement: Supplementary file 3 — Supplementary Figure 2 [file 12276_2018_142_MOESM3_ESM.tif]

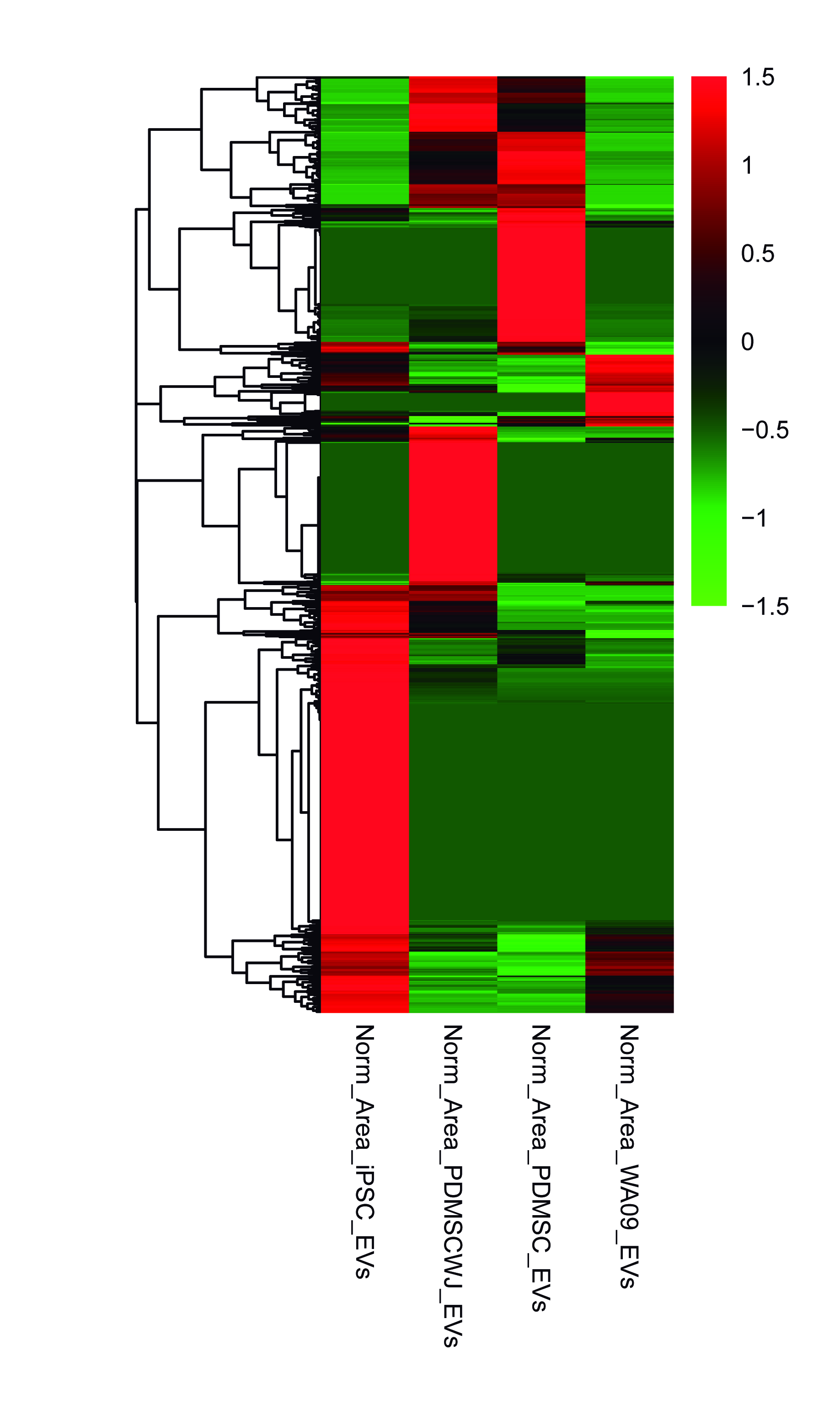

Supplement: Supplementary file 4 — Supplementary Figure 3 [file 12276_2018_142_MOESM4_ESM.tif]

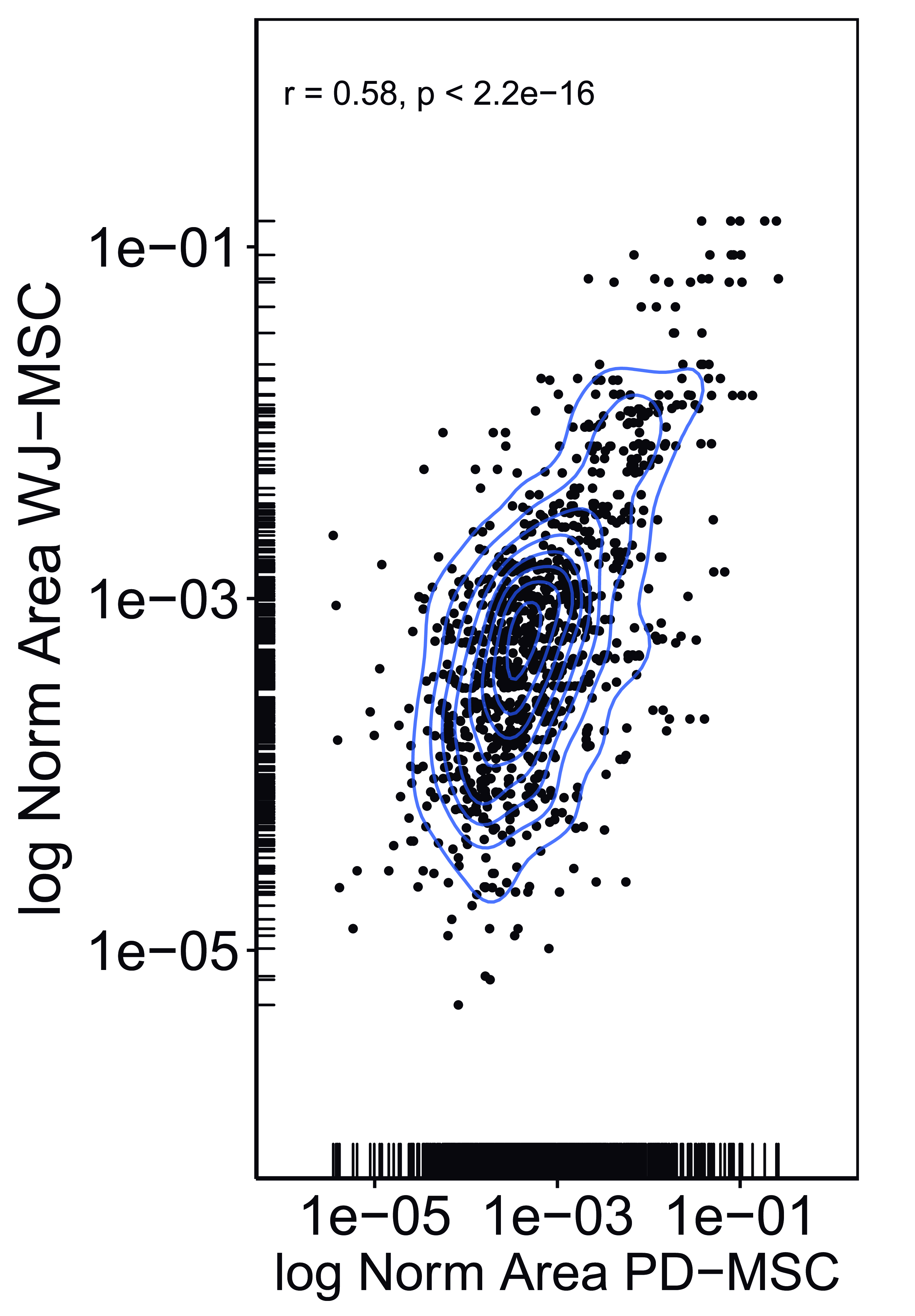

Supplement: Supplementary file 5 — Supplementary Figure 4 [file 12276_2018_142_MOESM5_ESM.tif]

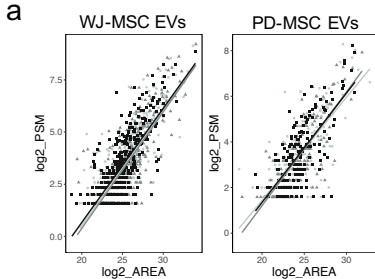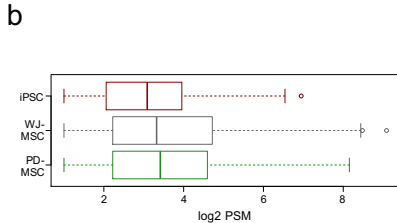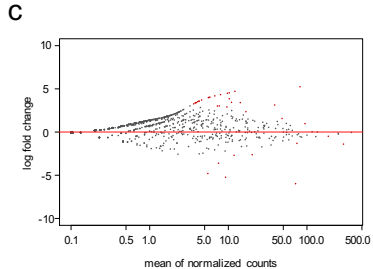

Supplement: Supplementary file 6 — Supplementary Figure 5 [file 12276_2018_142_MOESM6_ESM.pdf]

a

Number of hits in Cellular Component categories

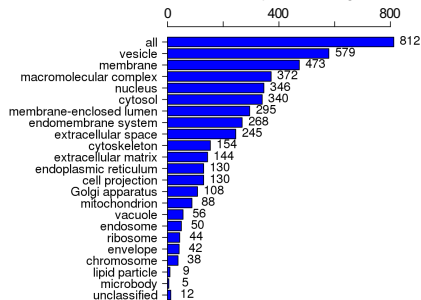

c

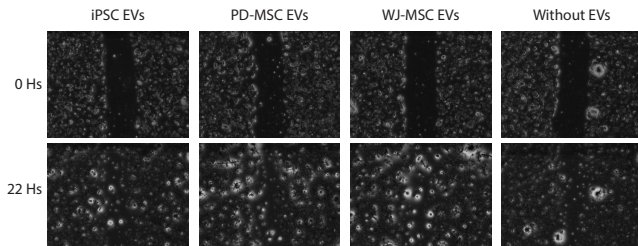

b

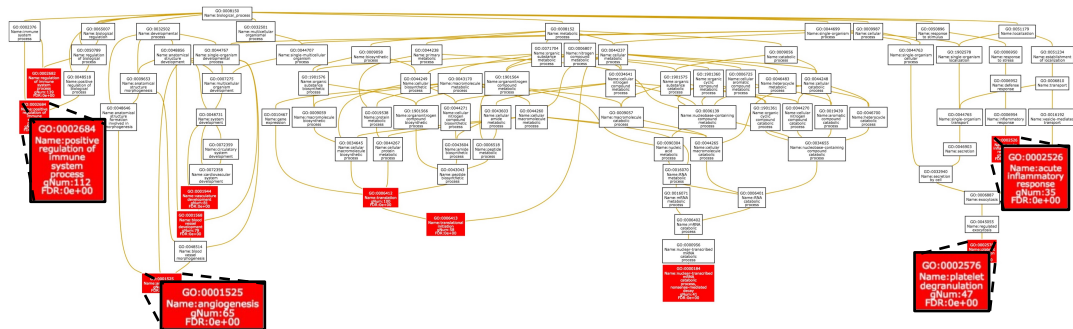

Supplement: Supplementary file 7 — Supplementary Figure 6 [file 12276_2018_142_MOESM7_ESM.pdf]
